# Protein of a thousand faces: The tumor-suppressive and oncogenic responses of p53

**DOI:** 10.3389/fmolb.2022.944955

**Published:** 2022-08-25

**Authors:** Mayra A. Marques, Guilherme C. de Andrade, Jerson L. Silva, Guilherme A. P. de Oliveira

**Affiliations:** Institute of Medical Biochemistry Leopoldo de Meis, National Institute of Science and Technology for Structural Biology and Bioimaging, National Center of Nuclear Magnetic Resonance Jiri Jonas, Federal University of Rio de Janeiro, Rio de Janeiro, RJ, Brazil

**Keywords:** tumor suppressor, oncogene, p53, aggregates, amyloid, structural biology

## Abstract

The p53 protein is a pleiotropic regulator working as a tumor suppressor and as an oncogene. Depending on the cellular insult and the mutational status, p53 may trigger opposing activities such as cell death or survival, senescence and cell cycle arrest or proliferative signals, antioxidant or prooxidant activation, glycolysis, or oxidative phosphorylation, among others. By augmenting or repressing specific target genes or directly interacting with cellular partners, p53 accomplishes a particular set of activities. The mechanism in which p53 is activated depends on increased stability through post-translational modifications (PTMs) and the formation of higher-order structures (HOS). The intricate cell death and metabolic p53 response are reviewed in light of gaining stability *via* PTM and HOS formation in health and disease.

## Introduction

**FIGURE 1:**
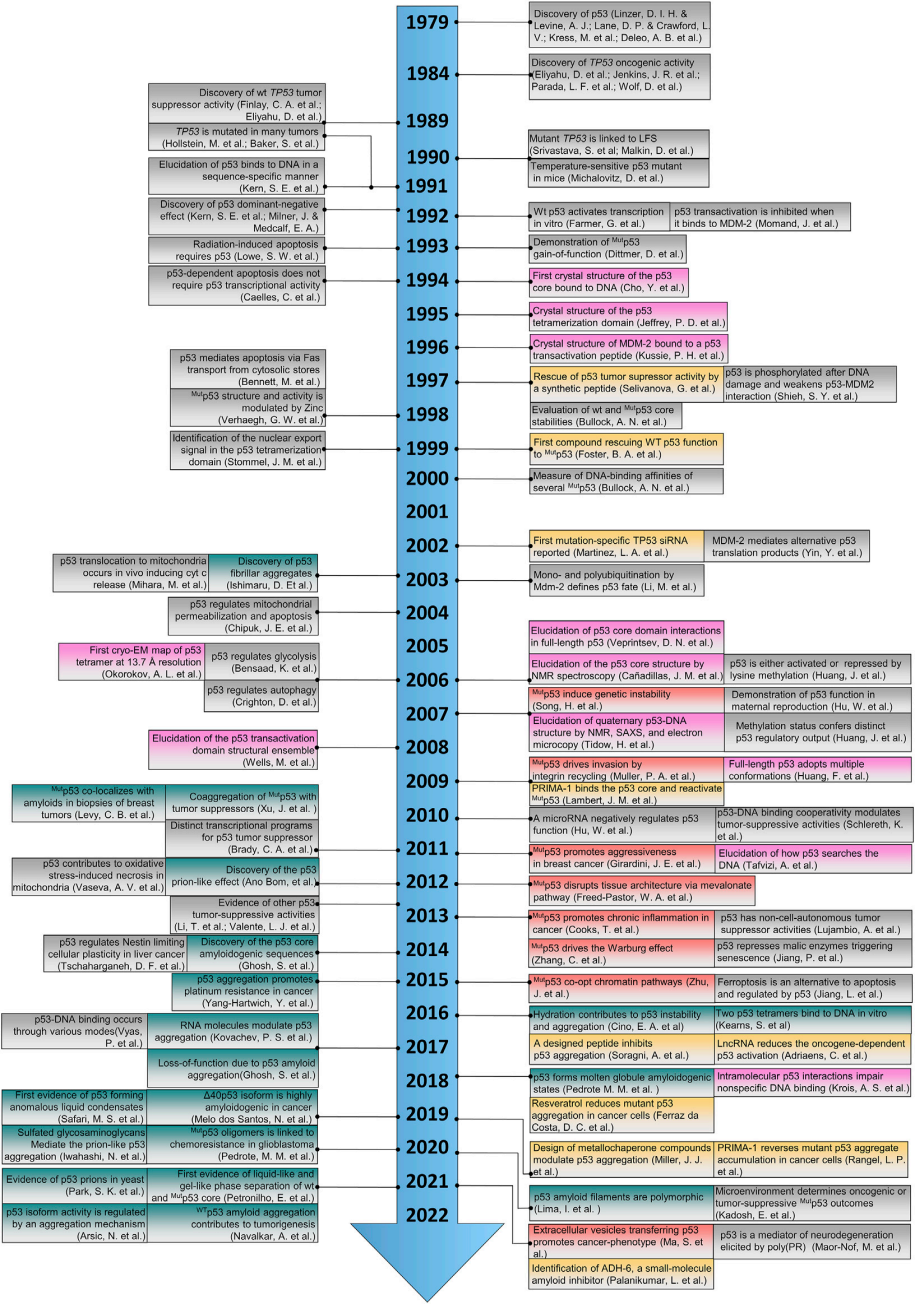
The timeline illustrates what the authors consider the most disruptive contribution in the p53 research over the last 40 years. Color coding shows seminal and breakthrough discoveries (gray), structural biology contribution (pink), therapeutic strategies (yellow), p53 gain-of-function examples (red), and p53-HOS research (green). We apologize to our colleagues in the p53 field whose work was not cited due to space limitations. References included in the timeline but not mentioned in the text: (Michalovitz et al., 1990; Farmer et al., 1992; Selivanova et al., 1997; Verhaegh et al., 1998; Foster et al., 1999; Stommel et al., 1999; Martinez et al., 2002; Yin et al., 2002; Lambert et al., 2009; Hu et al., 2010a; Adriaens et al., 2016; Kadosh et al., 2020; Maor-Nof et al., 2021).

Since the discovery of the tumor suppressor p53 back in the 80s (DeLeo et al., 1979; Kress et al., 1979; Lane and Crawford, 1979; Linzer and Levine, 1979), the biology of p53 has evolved dramatically (Figure 1). Initially recognized as the protector of the genome (Eliyahu et al., 1989; Finlay et al., 1989), p53 gained attention when wide-genome studies revealed the higher frequency of mutations within the TP53 gene (Baker et al., 1989; Hollstein et al., 1991). The follow-up of cancer cells and tumor tissues carrying TP53 mutations raised novel functionalities to p53 and the title of a pleiotropic regulator with oncogenic activity (Eliyahu et al., 1984; Jenkins et al., 1984; Parada et al., 1984; Wolf et al., 1984; Eliyahu et al., 1985; Michalovitz et al., 1986; Malkin et al., 1990; Srivastava et al., 1990). While some mutations affect the ability of p53 to bind DNA responsive elements (loss-of-function, LoF), others have a dominant-negative phenotype (Milner and Medcalf, 1991; Kern et al., 1992). The third group (gain-of-function, GoF) transforms the p53 into an oncogene (Dittmer et al., 1993). GoF p53 pursues cancer-promoting phenotypes through several mechanisms allowing tumor perpetration (Freed-Pastor and Prives, 2012; Muller and Vousden, 2013; 2014).

**FIGURE 2:**
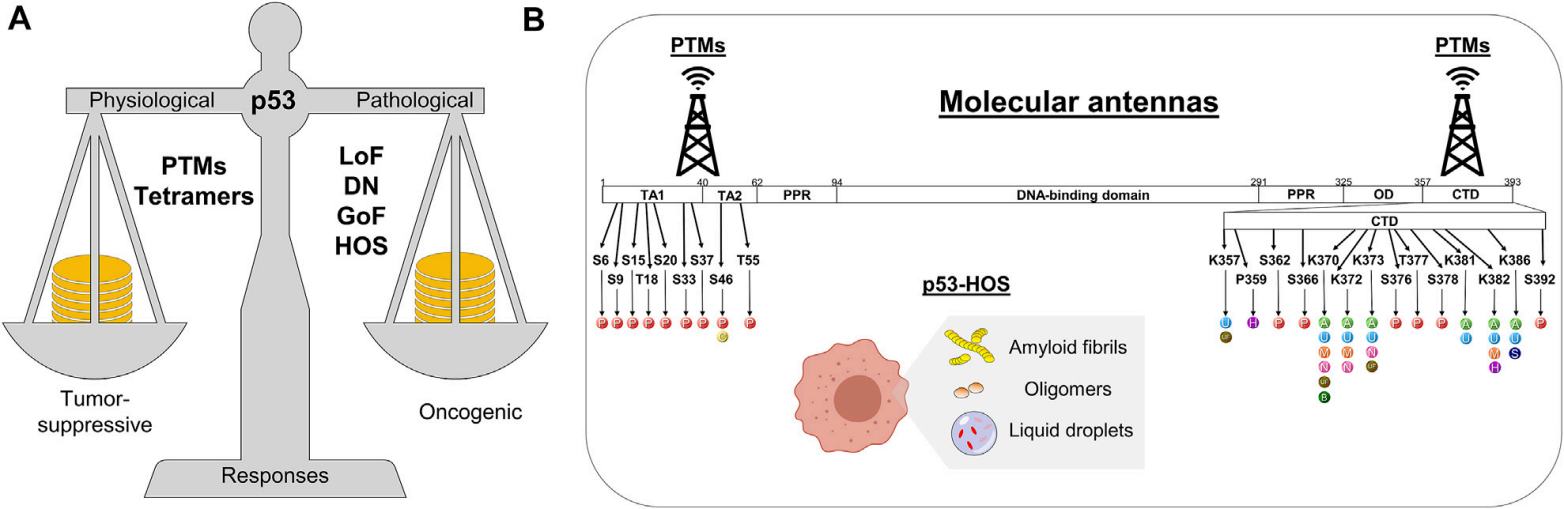
Schematics showing **(A)** the p53 balancing in heath and disease and **(B)** two mechanisms in which p53 is stabilized to execute transcriptional programs or gain-of-function activities. Residues that undergo post-translational modifications at the NTD and CTD are highlighted with their respective possible modification indicated as: P, Phosphorylation; C, Crotonylation; U, Ubiquitination; UF, UFMylation; H, Hydroxylation; A, Acetylation; M, Methylation; N, NEDDylation; B, β-hydroxybutyrylation; S, SUMOylation. PTMs, post-translational modifications; LoF, loss-of-function; DN, dominant negative; GoF, gain-of-function; HOS, higher-order structure; TA, transactivation domain; PPR, proline-rich motif; OD, oligomerization domain; CTD, C-terminal domain.

The efforts to decipher the p53 network happen at the physiological and pathological levels (Figure 2A) (Murray-Zmijewski et al., 2008; Vousden and Prives, 2009; Beckerman and Prives, 2010; Bieging et al., 2014; Pfister and Prives, 2017) and the list of p53 target genes continuously rises, making the p53 research a fascinating endeavor (Kastenhuber and Lowe, 2017). To some extent, the internal topology of p53 may help explain how it tunes tumor-suppressive or oncogenic responses (Joerger and Fersht, 2016). The p53 protein comprises the N-terminal transactivation domain (NTD or TAD), the DNA-binding domain (DBD), the oligomerization domain (OD or TET), and the C-terminal domain (CTD) (Figure 2B). The highly acidic NTD and the highly basic CTD undergo disorder-to-order states upon binding to cellular partners and are prone to post-translational modifications (PTM) (Brooks and Gu, 2003b; Bode and Dong, 2004; Kruse and Gu, 2008), affecting p53 stability, partner association, and signaling responses. These features make the p53 NTD and CTD work as molecular antennas (Figure 2B), tuned by conformational changes and PTM to deliver the most beneficial transcriptional program.

In addition, the tendency of wild-type and mutant p53 to form functional higher-order structures (HOS) (Ishimaru et al., 2003; Ano Bom et al., 2012; Silva et al., 2013; Ghosh et al., 2014; Silva et al., 2014; de Oliveira et al., 2015; Silva and Cordeiro, 2016; Ghosh et al., 2017; Pedrote et al., 2018; de Oliveira et al., 2019; de Oliveira et al., 2020; Lima et al., 2020; Pedrote et al., 2020; Navalkar et al., 2021; Petronilho et al., 2021) drives the tumor-suppressive vs. oncogenic responses (Figure 2B). The way p53 transform itself into these HOS *in vitro* and *in vivo* depends on the mutational status of p53 (Bullock et al., 1997; Levy et al., 2011), p53 cellular partners (Silva et al., 2010; Kovachev et al., 2017; Silva et al., 2022), a dysfunctional proteasome, an abnormal unfolded protein response (UPR), and an unbalance in p53 production. The overproduction of altered p53 combined with the exposure of amyloidogenic p53 segments (Ghosh et al., 2014) may lead to a panoply of p53-HOS. The classification of p53-HOS ranges from multimers (those larger than the active tetramer) (Stenger et al., 1992; Stenger et al., 1994; Kearns et al., 2016; Pedrote et al., 2020), to functional oligomers with amorphous or amyloid features (Ano Bom et al., 2012; Pedrote et al., 2020; Hibino et al., 2022), to more complex structures such as liquid condensates (Safari et al., 2019; Kamagata et al., 2020; Park et al., 2021; Petronilho et al., 2021) and amyloid filaments (Ishimaru et al., 2003; Galea et al., 2005; Ghosh et al., 2017; Lima et al., 2020) (Figure 2B). Unraveling the activities of p53-HOS is a recent and open field of exploratory research. Functional p53-HOS might represent a strategy in which wild-type and mutant p53 accomplish such a variety of unrelated activities as a tumor suppressor or an oncogene. This review outlines some roles of the p53 sentinel in cell death and metabolism and our group’s recent work showing the formation of p53-HOS and their potential implications for cancer perpetration.

## The p53 molecular antennas

Over 50 PTMs mediate p53 stability, activation, and gene selection, determining the cellular fate (Meek and Anderson, 2009; Carlsen and El-Deiry, 2021). Even though PTMs are present in DBD, the flexible NTD and CTD are the most frequent regions related to such modifications (Table 1). Phosphorylation is the prevalent PTM at the N-terminal, while the C-terminal is reversibly modified by phosphorylation, acetylation, ubiquitination, neddylation, and sumoylation (Appella and Anderson, 2001; Brooks and Gu, 2003a; Bode and Dong, 2004; Kruse and Gu, 2008).

**TABLE 1:** The molecular antennas: NTD and CTD p53 residues with PTM.

	Residue	PTM	Enzyme(s) responsible	Physiological effect	*In vivo*	*In vitro*	References
NTD	S6	P	CK1	p53 interaction with Smad2	Yes	Yes	Knippschild et al. (1997); Higashimoto et al. (2000); Meek and Anderson. (2009)
	S9	P	CK1	P53 interaction with Smad2	Yes	Yes	Knippschild et al. (1997); Higashimoto et al. (2000); Meek and Anderson. (2009)
	S15	P	ATM	Inhibit MDM2 binding	Yes	Yes	Shieh et al. (1997); Dumaz and Meek. (1999); Tibbetts et al. (1999); She et al. (2000); Saito et al. (2002); Shih et al. (2002)
			ATR	Cell apoptosis			
			DNAPK	Increased p53 transactivation activity			
			P38 kinase	Binding to p300			
			ERK				
	T18	P	CK1	Inhibit MDM2 binding	Yes	Yes	Dumaz et al. (1999); Sakaguchi et al. (2000); Teufel et al. (2009); Dai et al. (2019)
				Increased p53 binding to p300			
				Binding to Pellino1 and recruitment to DNA damage sites			
	S20	P	Chk2	Inhibit MDM2 binding	Yes	Yes	Hirao et al. (2000); She et al. (2002); Li et al. (2005)
			JNK1/2	Increased p53 stability			
			MAPKAPK2	Apoptosis			
			PLK-3				
	S33	P	P38 kinase	Apoptosis	Yes	Yes	Bulavin et al. (1999); Turenne and Price. (2001)
			GSK3β	Increased p53 transcriptional activity			
	S37	P	ATR	Inhibit MDM2 binding	Yes	Yes	Shieh et al. (1997); Tibbetts et al. (1999); Dohoney et al. (2004)
			DNAPK	Increased p53 transcriptional activity			
	S46	P	P38 kinase	Apoptosis	Yes	Yes	Bulavin et al. (1999); D’Orazi et al. (2001); Yoshida et al. (2006); Taira et al. (2007)
			HIPK2				
			PKCδ				
			DYRK2				
		C	Not identified yet	Inhibity p53 activity	Not reported	Yes	Liao et al. (2020)
	T55	P	ERK2	p53 activation; Stabilization/degradation	Yes	Yes	Yeh et al. (2001); Li et al. (2004); Jenkins et al. (2012)
			TAF1	Nuclear localization			
CTD	K357	U	Pirh2	P53 degradation	Yes	Yes	Leng et al. (2003)
				Decreased activity			
		UF	UBA5	Maintains p53 stability and tumor-suppressive activity	Yes	Yes	Liu et al. (2020)
			UFC1				
			UFL1				
	P359	H	PHD3	Decreased p53 ubiquitination	Yes	Yes	Rodriguez et al. (2018)
				Increased p53 stability			
	S362	P	IKK2	p53 destabilization		Yes	Xia et al. (2009a)
	S366	P	Chk2	Modulates p53 CTD acetylation	Yes	Yes	Ou et al. (2005); Xia et al. (2009b)
			IKK2	p53 destabilization			
	K370	A	P300/CBP	Increased p53 stability	Yes	Yes	Gu and Roeder, (1997)
				Increased p53transcriptional activity			
		U	MDM2	Degradation	Yes	Yes	Rodriguez et al. (2000)
				Nuclear export			
		M	Smyd2	Decreased p53 transcriptional activity	Yes	Yes	Huang et al. (2006)
		N	NEDD8	Inhibit p53 transcriptional activity	Yes	Yes	Xirodimas et al. (2004)
			MDM2-NEDD8 (*in vivo*)				
		B	CBP/p300	Decreases p53 acetylation and transcriptional activity	Yes	Yes	Liu et al. (2019)
		UF	UBA5	Maintains p53 stability and tumor-suppressive activity	Yes	Yes	Liu et al. (2020)
			UFC1				
			UFL1				
	K372	A	P300/CBP	Increased p53 stability	Yes	Yes	Gu and Roeder, (1997)
				Increased p53 transcriptional activity			
		U	MDM2	Degradation	Yes	Yes	Rodriguez et al. (2000)
				Nuclear export			
		M	Set9	Restricts p53 to the nucleus	Yes	Yes	Chuikov et al. (2004)
				Increased stability			
		N	NEDD8	Inhibit transcriptional activity	Yes	Yes	Xirodimas et al. (2004)
			MDM2-NEDD8 (*in vivo*)				
	K373	A	P300/CBP	Increased p53 stability	Yes	Yes	Gu and Roeder, (1997)
				Increased p53 transcriptional activity			
		U	MDM2	Degradation	Not reported	Yes	Rodriguez et al. (2000)
				Nuclear export			
		N	NEDD8	Inhibit transcriptional activity	Yes	Yes	Xirodimas et al. (2004)
			MDM2-NEDD8 (*in vivo*)				
		UF	UBA5	Maintains p53 stability and tumor-suppressive activity	Yes	Yes	Liu et al. (2020)
			UFC1				
			UFL1				
	S376	P^a^	PKC	Dephosphorylation promotes p53 binding to 14-3-3 proteins; Increased specific-DNA affinity	Yes	Yes	Waterman et al. (1998); Chernov et al. (2001)
	T377	P	LRRK2	Induces p21 expression	Not reported	Yes	Ho et al. (2015)
				Apoptosis			
	S378	P^a^	PKC	Dephosphorylation promotes p53 binding to 14-3-3 proteins; Increased specific-DNA affinity	Yes	Yes	Waterman et al. (1998); Chernov et al. (2001)
	K381	A	P300/CBP	Increased p53 stability	Yes	Yes	Gu and Roeder, (1997)
				Increased p53 transcriptional activity			
		U	MDM2	Degradation	Yes	Yes	Rodriguez et al. (2000)
				Nuclear export			
	K382	A	P300/CBP	Increased stability	Yes	Yes	Gu and Roeder. (1997); Rokudai et al. (2013)
			MOZ	Increased p53 stability			
				Increased p53 transcriptional activity			
		U	MDM2	Degradation	Not reported	Yes	Rodriguez et al. (2000)
				Nuclear export			
		M	SET8/PR-Set7	Decreased transcriptional activity	Yes	Yes	Shi et al. (2007)
		H	JMJD6	Decreases p53 acetylation and transcriptional activity	Yes	Yes	Wang et al. (2014)
	K386	A	P300/CBP	Increased p53 stability	Yes	Yes	Gu and Roeder, (1997)
				Increased p53 transcriptional activity			
		U	MDM2	Degradation	Yes	Yes	Rodriguez et al. (2000)
				Nuclear export			
		S	PIAS1	Nuclear export	Yes	Yes	Nie et al. (2021); Wen and Wang. (2022)
				Transcriptional activity inhibited			
	S392	P	PKR	Increased DNA-specific binding	Yes	Yes	Bayle et al. (1995); Sakaguchi et al. (1997); Bode and Dong. 2004); Castrogiovanni et al. (2018)
			FACT-CK2	Tetramerization			
			P38 kinase	Apoptosis			
DBD	K120	B	CBP/p300	Decreases p53 acetylation and transcriptional activity	Yes	Yes	Liu et al. (2019)
	E255	AR	PARP-1	p53 nuclear accumulation	Yes	Yes	Kanai et al. (2007)
	D256						
	E268						
PPR	S315	P	STK15	Increased p53 degradation	Yes	Yes	Katayama et al. (2004)
	K319	B	CBP/p300	Decreases p53 acetylation and transcriptional activity	Yes	Yes	Liu et al. (2019)
	K320	A	PCAF	Modulates p53 affinity to DNA	Yes	Yes	Liu et al. (1999)
	K320	N	FBXO-11	Inhibit p53 transcriptional activity	Yes	Yes	Abida et al. (2007)
	K321						
OD	R333	M	PRMT5	Oligomerization	Not reported	Yes	Jansson et al. (2008)
	R335			Translocation to the nucleus			
	R337			G1 arrest			

a Constitutively phosphorylated in unstressed cells.

P, Phosphorylation; C, Crotonylation; U, Ubiquitination; UF, UFMylation; H, Hydroxilation; A, Acetylation; AR, ADP-Rybosilation; M, Methylation; N, NEDDylation; B, β-hydroxybutyrylation; S, SUMOylation; ATM, ataxia telangiectasia mutated; ATR, ataxia telangiectasia and Rad3 related; CBP, CREB binding protein; CK1, casein 1 kinase; Chk2, checkpoint kinase 2; DNAPK, DNA-dependent protein kinase; DYRK2, dual-specificity tyrosine-phosphorylation-regulated kinase 2; ERK, extracellular signal-regulated kinase; ERK2, Extracellular Signal Regulated Kinase 2; FACT-CK2, casein kinase 2 (CK2) and the chromatin transcriptional elongation fator FACT (a heterodimer of hSpt16 and SSRP1); GSK3β, Glycogen Synthase Kinase (GSK3) Beta; HIPK2, Homeodomain-interacting protein kinase-2; IKK2, IB kinase 2; JMJD6, Jumonji domain-containing 6; JNKs, c-Jun NH2-terminal kinases; LRRK2, Leucine-rich repeat kinase 2; MAPKAPK-2, MAPK Activated Protein Kinase 2mitogen-activated protein (MAP) kinases; MDM2, oncoprotein murine double minute-2; MOZ, monocytic leukemic zinc finger (MOZ) histone acetyltransferase; NEDD8, NEDD8 Ubiquitin Like Modifier; PARP-1, Poly(ADP-ribose) polymerase 1; PCAF, P300/CBP-associated fator; PHD3, Prolyl-4-hydroxylase domain 3; PIAS1, Protein inhibitor of activated STAT-1; Pirh2, Ubiquitin ligase with RING-H2 domain; PKCδ, Protein kinase C delta; PKR, double-stranded RNA activated protein kinase; PLK-3, Polo-like kinase 3; PRMT5, Protein arginine methyltransferase 5; SET8/PR-Set7, SET-domain containing protein 8; Set9, Histone-lysine N-methyltransferase set9; Smyd2, SET And MYND Domain Containing 2; STK15, serine/threonine kinase-15 TAF1, Transcription initiation factor TFIID subunit 1; UBA5, E1- and E2-like enzymes ubiquitin-like modifier activating enzyme 5; UFC1, ubiquitin-fold modifier-conjugating enzyme 1; UFL1, E3-like ligase UFM1-specific ligase 1.

At least 13 serines (Ser) and three threonines (Thr) outside the DBD and the TET are phosphorylated (Kruse and Gu, 2008; Carlsen and El-Deiry, 2021; Wen and Wang, 2021). P53 phosphorylation usually increases p53 stability or modulates its interactions with molecular partners. Serines at positions 6, 9, 15, 20, 33, 37, and 46 and threonines at positions 18 and 81 are phosphorylated after different stressors, including exposure to UV light or ionizing radiation (Appella and Anderson, 2001; Kruse and Gu, 2008). Ser15, for example, is phosphorylated by ataxia telangiectasia-mutated kinase (ATM) and ataxia telangiectasia and rad3-related kinase (ATR), leading to cell apoptosis (Tibbetts et al., 1999; Saito et al., 2002). After DNA damage, the DNA-dependent protein kinase (DNA-PK) phosphorylation reduces p53 interaction with the oncoprotein murine double minute-2 (MDM2), *in vivo* and *in vitro*, preventing p53 degradation (Shieh et al., 1997). She et al. showed that UVB-induced phosphorylation by extracellular signal-regulated protein kinases (ERKs) and p38 kinase leads to apoptosis (She et al., 2000). Phosphorylated Ser15 is also critical for p53 transactivation, mainly through increased binding to p300 (Dumaz and Meek, 1999). In MCF7 cells, UV-induced phosphorylation at Ser15 and Ser37 was critically dependent on prior phosphorylation at Ser33 and Ser46 by p38 kinase (Bulavin et al., 1999). After DNA damage, phosphorylation at Ser15 occurs *in vivo* and *in vitro* before Thr18 phosphorylation, leading to p53-MDM2 disruption (Sakaguchi et al., 2000). Phosphorylation at Thr18 was also reported to increase binding to p300 (Teufel et al., 2009), and more recently, pThr18 revealed p53 binding to Pellino1, an E3 ubiquitin ligase, inducing p53 translocation to DNA damage sites (Dai et al., 2019). Thr18 and Ser20 showed significant phosphorylation in human breast cancer expressing wild-type p53 (Craig et al., 1999). While UV light promotes JNKs and MAP kinases to phosphorylate Ser20, leading to apoptosis (She et al., 2002), Chk2 phosphorylation at Ser20, in response to ionizing radiation, interferes with p53-MDM2 binding (Hirao et al., 2000). Doxorubicin causes Thr55 phosphorylation by ERK2 (Yeh et al., 2001), and it is also a TAF1 phosphorylation target critically related to p53 degradation in unstressed cells. Interestingly, the pThr55 status reduces in response to DNA damage (Li et al., 2004).

Phosphorylation at Ser392 was reported to increase DNA-specific binding, inducing tetramer formation and p53 mitochondrial translocation leading to transcription-independent apoptosis (Bayle et al., 1995; Sakaguchi et al., 1997; Castrogiovanni et al., 2018). Experiments using a p53 phosphorylation mimetic S392E showed increased thermal stability and affinity to non-specific DNA (Nichols and Matthews, 2002). Moreover, a phosphopeptide assay demonstrated that Ser392 phosphorylation by IKKβ mediates p53 activation after glutamine deprivation (Ishak Gabra et al., 2018). Waterman et al. demonstrated that Ser376 and Ser378 are constitutively phosphorylated in unstressed cells, and dephosphorylation of Ser376 caused by ionizing radiation promotes p53 binding to 14-3-3 proteins and increases its affinity to sequence-specific DNA (Waterman et al., 1998). Phosphorylation at Ser315 stimulated by STK15 increases p53 degradation induced by Mdm2-ubiquitination and facilitates tumorigenesis (Katayama et al., 2004). Unphosphorylation at Ser362 and Ser366 has an essential role in p53, increasing p53 stability, modulating p21 expression, and altering the G1 cell cycle (Xia et al., 2009).

p53 was the first non-histone and transcription factor regulated by acetylation of histone acetyltransferases (HAT). Lys370, Lys372, Lys373, Lys381, Lys382, and Lys386 are modified by receiving an acetyl group caused by p300**/**CBP, which influences p53 activity and DNA binding (Gu and Roeder, 1997). PCAF was also reported as an acetylase at Lys320 in response to DNA damage, modulating p53 affinity to DNA (Liu et al., 1999). In mice, acetylation at Lys317 (Lys320 in humans) causes decreased p53 apoptotic activity in response to DNA damage (Chao et al., 2006). Deacetylation of p53 by HADC1, –2, or –3 decreases its transcriptional activity (Juan et al., 2000; Luo et al., 2000). SIRT1 deacetylates p53 at Lys382, reducing p53 transcriptional activity, cell cycle arrest, and apoptosis (Vaziri et al., 2001). SIRT1 activity is modulated by AMPK kinase and is inactivated after phosphorylation. It leads to enhanced p53 acetylation and apoptosis in hepatocellular carcinoma (Lee et al., 2012). In colorectal cancer (CRC), ArhGAP30, a Rho GTPase-activating protein, promotes p300 binding to p53 C-terminal and is critical for p53 acetylation (Wang J. et al., 2014).

Methylation at Lys372 by Set9 methyltransferase restricts p53 to the nuclear localization and increases its stability (Chuikov et al., 2004). Methylation at Lys370 by Smyd2 repress p53 transcriptional activity. Because the p53-Smyd2 interaction is blocked during Lys372 methylation, the Lys370 site is repressed by methylation at Lys372. In such a case, methylation is essential for activating and inhibiting p53 (Huang et al., 2006). LSD1 is the demethylase responsible for mono or dimethylation at Lys370; while Lys370 monomethylation represses p53 activity, Lys370 dimethylation leads to 53BP1 interaction. Because LSD1 prefers to demethylate Lys370, it inhibits p53 transcriptional activity and apoptosis (Huang et al., 2007). In testicular teratocarcinoma cells, p53 is repressed by methylation at Lys370 and Lys372 by Smyd2 and Set9, respectively (Zhu et al., 2016). The SET8/PR-Set7 promotes monomethylation at Lys382, repressing the p53 transcriptional activity and decreasing pro-apoptotic function (Shi et al., 2007). Jansson et al. revealed p53 arginine (Arg) methylation by PRMT5 in DNA damage response, affecting its target gene specificity. Methylation at Arg333, Arg335, and Arg337 promotes p53 oligomerization, translocation to the nucleus, and G1 arrest (Jansson et al., 2008).

Ubiquitination is part of the p53 regulatory axis: in unstressed cells, p53 levels are tightly regulated by ubiquitin-binding, triggering proteolysis. MDM2 ubiquitinates Lys370, Lys372, Lys373, Lys381, Lys382, and Lys386, which leads to proteasome-dependent degradation. Although ubiquitin is bound to the CTD, the NTD is required for p53-MDM2 interaction (Rodriguez et al., 2000; Chao, 2014). Low MDM2 activity leads to p53 monoubiquitination, promoting p53 nuclear extrusion. Contrastingly, when MDM2 activity is high, p53 polyubiquitination leads to nuclear degradation (Li et al., 2003). Some other E3-ligases are essential regulators of p53 ubiquitination, such as Arf-BP1, COP1, Pirh2, MSL2, Parc, TARF7, and Cullin 4B, while deubiquitination enzymes such as HAUSP, OTUB1, USP10, USP11, OTUD1, and ATX regulate cell’s p53 level (Chen et al., 2006; Hakem et al., 2011; Halaby et al., 2013; Kwon et al., 2017; Pan and Blattner, 2021).

Ubiquitination-like modifications are reported in p53 regulation as well. MDM2 mediates anchoring of NEDD8 to p53 at Lys370, Lys372, and Lys373. The conjugation with NEDD8 leads p53 to decreased transcriptional activity. FBXO-11 also promotes this type of modification, specifically at Lys320 and Lys321 *in vitro* and *in vivo* (Xirodimas et al., 2004; Abida et al., 2007)*.* The small ubiquitin-like modifier (SUMO) protein is another example of a ubiquitin-like modification that p53 undergoes at Lys386. In response to DNA damage and oxidative stress, SUMOylation mediates p53 nuclear export and regulates p53 transcriptional activity, with a critical role in preventing tumorigenesis (Santiago et al., 2013; Scurr et al., 2017; Wen and Wang, 2021). The discovery of a novel PTM called ubiquitin-fold modifier 1 (UFM1) showed to antagonize ubiquitination: UFM1-ligase promotes UFMylation and competes with MDM2 for p53 binding. UFMylation maintains p53 stability and tumor-suppressive activity in contrast to ubiquitination, which leads to proteasomal degradation (Liu et al., 2020).

Moreover, Poly(ADP-ribose) polymerase 1 (PARP-1) promotes ADP-ribosylation at Glu255, Asp256, and Glu268 *in vivo*, causing p53 nuclear accumulation by blocking the p53-Crm1 interaction, a nuclear export receptor (Kanai et al., 2007). Interestingly, PARP-1-induced ADP-ribosylation has shown to modulate phase separation and transition in several proteins (Leung, 2020). Some other PTMs are still coming to light in the last few years. Hydroxylation at Pro359 by PHD3 inhibits p53 association with deubiquitinating enzymes USP7 and USP10, a condition that reduces p53 ubiquitination and increases p53 stability (Rodriguez et al., 2018). Hydroxylation at Lys382 by Jumonji domain-containing 6 (JMJD6) decreases p53 acetylation and transcriptional activity by promoting its association with MDMX (Wang et al., 2014a). β-Hydroxybutyrylation at Lys120, Lys319, and Lys370 reduces p53 acetylation and decreases transcription of p53-dependent genes (Liu et al., 2019); crotonylation at Ser46 seems to inhibit p53 activity, mainly increasing p53-dependent glycolytic activity and cancer cell proliferation upon DNA damage or metabolic stress (Liao et al., 2020).

Besides playing different regulatory roles, PTMs may also induce p53 conformational changes. For instance, phosphorylation at Thr18 alters the MDM2 interacting α-helix structure within the p53 NTD. Phosphorylation of threonine-proline motifs within the proline-rich region allows PN1 prolyl-isomerase binding and peptidyl-prolyl isomerization leading to reduced p53 affinity to MDM2 and increased p300/CBP affinity (Toledo and Wahl, 2006). Kar et al. proposed that p53 phosphorylation at the NTD induces open conformations, allowing p53 interaction with p300/CBP transcription factors (Kar et al., 2002). Phosphorylation at Thr55 leads to structure modifications at the NTD, stabilizing NTD-DBD interactions to form tetramers and reducing DNA affinity (Sun et al., 2020). Acetylation also triggers p53 conformational changes to more open states (Reed and Quelle, 2015). For example, acetylation of K120 leads to L1 loop expansion, stimulating sequence-specific DNA binding (Vainer et al., 2016)

The p53 NTD and CTD help p53 transmit and receive signals through PTMs. Even if different modifications act with opposite responses, the complete inhibition or activation of p53 transcriptional activity may require multiple PTMs. P53 PTMs provide complex and combinatorial regulation, enabling the protein to recruit many partners. Of note, the PTMs’ role in protein oligomerization is somewhat puzzling. Likely, PTMs alone are not responsible for shifting populations towards higher-order states, though PTMs appear to recruit accessory proteins that induce or prevent multimeric assembly. For instance, acetylation within the C-terminal lysine-rich domain has been implicated in tetrameric packing by recruiting the 14-3-3 protein family (Itahana et al., 2009). Interestingly, acidic domain-containing proteins are recruited to interact with p53 in acetylation and charge-dependent fashion, controlling transcription activity (Wang et al., 2020). Besides, p53 lysine acetylation creates a docking site for acidic domain-containing proteins (Wang et al., 2017). P53 acetylation defect decreases stability and impairs transactivation. For instance, the SET protein inhibits p53 transcriptional activity in rest conditions. After DNA damage, the positive charge of lysine sidechains is neutralized upon acetylation, thereby eliminating the docking site for the acidic-domain-containing regulators. SET depletion in tumor cells reactivates p53 function and controls tumor growth, evidencing a possible therapeutic target (Wang et al., 2016). Further, ubiquitination recruits Ubc13 which increases p53 stability but prevents its tetramerization, trapping p53 at the cytoplasm and attenuating its transcriptional activity (Laine et al., 2006; Topisirovic et al., 2009). Indeed, much more investigation is required to elucidate the mechanism of PTMs-induced high order assembly in p53.

## The p53-higher-order structures

Protein hubs like p53 comprise intrinsically disordered regions (IDRs) that are common targets for PTMs. Additionally, the IDR plasticity enables conformational diversity, leading to a multiplicity of functions pertinent to cell survival (Jeong et al., 2001; Oldfield et al., 2008). Thus, challenging the “gene-protein-structure-function” paradigm and composing the “gene-protein-conformational ensemble-function diversity” scenario, p53 operates an intricate role beyond the classical view of a tumor suppressor.

P53 exists as a mixture of monomers, dimers, and tetramers (Rajagopalan et al., 2011). Within a living cell under basal conditions, p53 dimers are favored, but after DNA damage, most p53 undergoes a tetrameric arrangement (Gaglia et al., 2013). P53 dimers-of-dimers form a tightly packed tetramer *via* intermolecular β-sheets that regulate transcriptional and tumor-suppressive activity (Shaulian et al., 1992; Jeffrey et al., 1995; Bode and Dong, 2004). The transcriptional activity of p53 requires binding to specific DNA responsive elements (REs). The p53 RE comprises two copies of a palindromic 5′RRRCWWGYYY3′ (where R = A/G, W = A/T, Y = C/T) half-sites spaced by 0–13 base pairs (Kern et al., 1991; el-Deiry et al., 1992; Vyas et al., 2017; Cai et al., 2019; Silva et al., 2022). Molecular models of the crosslinked p53DBD-DNA complex revealed p53 tetramers interacting with two REs (Cho et al., 1994). However, single-molecule studies showed that p53 tetramers could bind DNA containing only a half-site and have different spacing between half-sites (Ly et al., 2020). Notwithstanding, the distribution of these p53 complexes and their kinetic stabilities are impacted compared with canonical REs (Ly et al., 2020). This finding is consistent with full-length p53 micrographs showing octamers organized as a pair of tetramers, suggesting that two p53 tetramers can bind each half-site to form octamers (Stenger et al., 1992; Stenger et al., 1994; Kearns et al., 2016).

The DBD of p53 is highly conserved among other species revealing that DNA-binding is vital for p53 physiology (Brandt et al., 2009; Levine, 2020). Missense mutations of the *TP53* gene are mainly found within the exons encoding the DBD (Hollstein et al., 1991), leading to the generation of non-native structures implicated in cancer (Joerger et al., 2006; Baugh et al., 2018). Non-native p53 undergoes considerable structural modifications (Ishimaru et al., 2003; Ishimaru et al., 2003; Ishimaru et al., 2004; Ang et al., 2006; Joerger et al., 2006; Joerger and Fersht, 2007; Bom et al., 2010; Pedrote et al., 2018) and as a multifunction and cooperative protein, the effects of a single point mutation could disrupt an entire network hub.

The rearrangement of p53 due to the loss of inter and intramolecular regulatory contacts illustrates Dr. Jekyll and Mr. Hyde (Silva and Cordeiro, 2016). In this scenario, one p53-HOS found by our group is p53 amyloid fibrils (Ishimaru et al., 2003; Ano Bom et al., 2012). Ishimaru et al. modulated high hydrostatic pressure (HHP) and sub-zero temperatures to isolate a metastable state of p53-DBD. The primary modifications were clustered within the DNA-binding region and surrounding the hydrophobic core, sites maintaining native contacts (Ishimaru et al., 2003). Moreover, the p53 DBD adopts non-native intermediates required for amyloid-like fibrils formation (Ano Bom et al., 2012; Ghosh et al., 2017; Pedrote et al., 2018).

It is notorious that protein function and interaction specificity are conserved throughout evolution, impacting the distribution of protein conformations. Therefore, p53 requires marginally stable conformations to exert its function (Canadillas et al., 2006; Cino et al., 2016). Consequently, local violations drive the conserved yet inherently metastable DBD toward specific conformational states that are likely to fuel the aggregation process, triggering tumor formation.

A clear relationship between protein misfolding, aggregation, and clinical reports has been established in human disorders (Soto and Pritzkow, 2018). In cancer, such mechanisms are largely unknown. Our results reveal that p53-DBD and p53-DBD-R248Q are prone to aggregate *in vitro* as amyloid fibrils (Ishimaru et al., 2003). Interestingly, the mutant shows a prion-like behavior, accelerating wild-type p53-DBD aggregation (Ano Bom et al., 2012; Ghosh et al., 2017; Iwahashi et al., 2020; Navalkar et al., 2021; Park et al., 2021). The prionoid characteristics include pathological misfolding, template conversion, aggregation, and self-propagation. Seminal articles highlight the presence of mutant p53 aggregates in tumor cells (Levy et al., 2011; Xu et al., 2011; Ano Bom et al., 2012; Yang-Hartwich et al., 2015; Pedrote et al., 2020), the cell-to-cell p53 transmissibility (Forget et al., 2013; Ghosh et al., 2017; Navalkar et al., 2021), and the recovery of the native phenotype by pharmacological inhibition of the p53 aggregation (Soragni et al., 2016; Ferraz da Costa et al., 2018; Silva et al., 2018; Miller et al., 2019; Rangel et al., 2019; Palanikumar et al., 2021). P53 isoforms were recently shown to aggregate in cancer cells (Melo Dos Santos et al., 2019) and control invasiveness (Arsic et al., 2021).

The three-dimensional structure of a functional p53-HOS is still unknown. A functional HOS assumed by p53 occurs when the p53 C-terminus interacts with the tyrosine kinase c-Abl (Nie et al., 2000). This interaction is necessary for the transactivation of genes involved with tumor suppression and cell growth, and deletion of the c-Abl-p53 interacting regions inhibits their antiproliferative activities (Nie et al., 2000). Further, mutant p53 aggregates can sequester either wild-type p53 or c-Abl into oligomeric forms in triple-negative breast cancers, thereby exerting a dominant-negative effect (Morrison et al., 2017). Again, the exact atomic organization of this protein cluster is unknown, but once elucidated, it would be a potent target for pharmacological inhibition.

Additionally, recent evidence suggests the participation of p53 in liquid-liquid phase separation (LLPS) (Safari et al., 2019; Kamagata et al., 2020; Park et al., 2021; Petronilho et al., 2021). This event may be linked to transcriptional regulation of p53 (de Oliveira et al., 2019; de Oliveira et al., 2020). IDRs are often required to assemble supramolecular structures into condensates that serve as a scaffold for preserving and activating the DNA transcription machinery (Hnisz et al., 2017; Shrinivas et al., 2019). Recent evidence shows that exogenous mutant p53 forms liquid droplets in the nucleolus of carcinoma cell lines, opening opportunities for studying the mechanism of oncogenic gain-of-function (Petronilho et al., 2021). Further, not only full-length p53 but also the p53-DBD undergoes phase separation and aggregation, showing that the p53 most conserved region carries inherent properties to HOS formation (Petronilho et al., 2021). Do several questions still lack answers: 1) are there functional aspects of p53 in LLPS? 2) would mutant p53 drive this arrangement? 3) Is there a favorable cellular condition for this supramolecular assembly?

An atomic-level investigation is needed to reveal the high-resolution structure of the full-length wild-type or mutant p53 bound to DNA or other cellular partners. There is still an even more significant structural gap regarding oligomeric or fibrillar assemblies, besides the new molecular arrangement of p53 condensates in liquid droplets. High-resolution structures of this essential multifunctional protein and its partners are required to understand the physiological and pathological mechanisms of p53.

## How to visualize the p53 structure

Elucidating the conformational ensemble of full-length p53 at near-atomic resolution remains a challenge. The first report of a p53 structure unraveled how the p53 DBD binds to the DNA (Cho et al., 1994), and was followed by two additional p53 domains: the tetramerization domain, TET (Jeffrey et al., 1995), and the transactivation domain, TAD (Kussie et al., 1996). Nuclear magnetic resonance (NMR) data revealed the dynamic behavior of p53, shedding light on its conformational continuum (Canadillas et al., 2006; Veprintsev et al., 2006).

A Pubmed search of full-length p53 and cryo-electron microscopy (or Cryo-EM) renders few results (Okorokov et al., 2006; Tidow et al., 2007; Melero et al., 2011; Liou et al., 2021). The molecular envelope of the DNA bound to p53 obtained from electron micrographs reveals at least four distinct conformations for the p53-DNA complex (Melero et al., 2011). Advances in hardware, software, and direct detectors led to the “resolution revolution” in cryo-EM, allowing an increasing number of published structures every year (Subramaniam et al., 2016; Marques et al., 2019; Benjin and Ling, 2020). A recent work elucidated the atomic model of the human p53/Pol II complex at 4.6 Å resolution using cryo-EM (Liou et al., 2021). According to the data, p53 interacts with Pol II as a monomer, suggesting that Pol II’s “unique positioning of the p53 core domain may provide both a geometric capability for RE recognition and a novel position for Pol II to interact with target p53 gene promoters” (Liou et al., 2021).

In addition, cryo-EM brought to light structures of large complexes, previously unimaginable if based only on X-ray diffraction. Thus, supramolecular complexes like viruses, amyloid filaments (Kollmer et al., 2019; Wu et al., 2021), and bacterial appendages are now more easily solved at near-atomic resolution. Electron microscopy of p53 amyloid fibrils (Ghosh et al., 2017; Lima et al., 2020) suggests that cryo-EM is suitable for solving p53 amyloid filaments and p53-HOS, such as oligomers and the tetrameric p53-DNA complex, but such structures have been not published yet.

The goal to achieve the highest p53 resolution possible has the challenge of p53 presenting labile segments. Therefore, elucidating the full-length p53 structure at near-atomic resolution will demand combined techniques and a solid biochemistry design. One strategy would be trapping the p53 labile NTD and CTD by complexing p53 with cellular partners (Melero et al., 2011; Liou et al., 2021), such as nucleic acids. Even more intriguing is the apparent molecular p53 chameleon characteristic. *In silico* experiments have shown that a p53 CTD motif comprising residues 374 to 388 distinguishes four different p53 partners depending on their conformational assembly. These residues exhibit distinct secondary structures in each complex, assuming α-helices, β-sheets, and coiled-coil (Uversky, 2016). It shows that p53 structural specificity guides different partner binding, corroborating that assembly modulation is critical for understanding p53 activities.

Molecular dynamics revealed that the CTDs in p53 tetramers directly interact with DNA *via* nonspecific electrostatic interactions (Demir et al., 2017). P53 constructs comprising the TET + CTD showed that p53 translocates into DNA much faster than the full-length p53, indicative of the NTD regulatory role (Tafvizi et al., 2011). Surprisingly, when the p53 CTD is withdrawn, there is no p53 movement along with the DNA. The mechanism states that p53 interacts with DNA in a searching-recognition fashion (Tafvizi et al., 2011). First, the CTD searches the DNA through nonspecific and dynamic interactions, followed by high-affinity and long-lasting binding mediated by the DBD (Tafvizi et al., 2011). Models arising from X-ray diffraction showed the tetrameric p53-DBD region interacting with DNA and the truncated form of the p53-DBD-TET with p21 and DNA (Emamzadah et al., 2011).

Mapping p53 intramolecular interactions are indispensable for therapeutic strategies (Wang and Fersht, 2017; Krois et al., 2018; Melo Dos Santos et al., 2019). Recently, attempting to elucidate high-resolution structures using artificial intelligence, a group of researchers developed software capable of predicting the three-dimensional biomolecule arrangement from its primary sequence (Senior et al., 2020). Despite the success in predicting structures with well-defined folding, proteins with labile segments or participating in macromolecular complexes remain a significant challenge but harbor enormous potential. Therefore, to achieve high resolution, the pillars of structural biology might be complementary: X-ray diffraction, cryoelectron microscopy (Cryo-EM), nuclear magnetic resonance (NMR), molecular dynamics, and computational predictions.

NMR is pivotal for unraveling the dynamic behavior between p53 and cellular partners, the high-energy intermediates (Wells et al., 2008), and molten globule amyloidogenic states (Bom et al., 2010; Pedrote et al., 2018). Krois et al. demonstrated the interaction between the DBD and the NTD through electrostatic interactions. This assembly increases the p53 stability in the solution (Krois et al., 2018). Moreover, the NTD regulates the interaction between DBD and specific DNA sequences. Interestingly, the comparison between DBD and NTD-DBD constructs *in vitro* revealed the propensity of the DBD to form aggregates, which is ceased with the presence of the NTD region (Melo Dos Santos et al., 2019). An increased p53 amyloid aggregation is present in endometrium carcinoma cell lines expressing a truncated p53 isoform at the NTD (Δ40), confirming that the p53 NTD has an intramolecular downregulatory role on p53 aggregation (Melo Dos Santos et al., 2019). These results point to a possible role of conformational control and protection of metastable regions (Cino et al., 2016; Melo Dos Santos et al., 2019).

The description of p53-DBD ensembles is scarce given the instability of the DBD, which often leads to aggregation during NMR data acquisition. However, other regions such as the TET, CTD, and NTD confers stability to the tetrameric protein. The rational design of p53 mutations stabilizing the DBD is an alternative for dealing with such a level of domain instability (Joerger et al., 2004). Analyzing the full-length p53 could help understand the regulatory role of specific segments for amyloid aggregation, phase transition, and liquid droplet formation. Using particular pulse sequences and experimental datasets for large complexes could reveal the molecular dynamics and tumbling at different time scales, helping to clarify various conformational changes that p53 may take place (Fawzi et al., 2011; Venditti et al., 2016; Tugarinov et al., 2022).

## The pro-death and pro-survival p53 tumor-suppressive response

**FIGURE 3:**
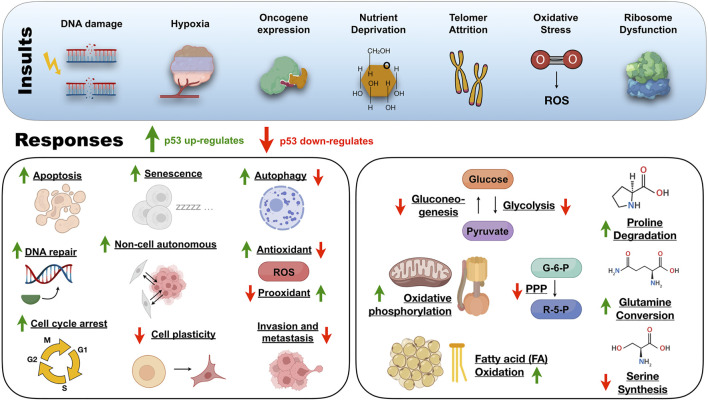
An overview of the insults and responses triggered by the protein of a thousand faces under physiological circumstances. ROS, reactive oxygen species; PPP, pentose phosphate pathway; G-6-P, glucose-6-phosphate; R-5-P, ribose-5-phosphate.

Unraveling the p53 structural ensemble in a test tube is far from what is happening inside stressed cells. Therefore, a comprehensive idea of how p53 works from the structural and cellular viewpoint would help clarify the role wild-type and mutant p53 assume in health and disease (Vousden and Lane, 2007). The p53 transcriptional program is context-dependent to safeguard the genome and does not require full p53 activation (Jiang D. et al., 2011). P53 triggers a specific program depending on the stressor level, the target gene promoter architecture, cofactor recruitment, the level of p53 expression, and the p53 PTM status. Signals such as DNA damage, hypoxia, oncogene expression, nutrient deprivation, telomere attrition, oxidative stress, and ribosome dysfunction activate p53 by enhancing the protein stability or through PTM (Bieging et al., 2014) (Figure 3). The p53-driven tumor-suppressive response deals with a broad cohort of target genes (Brady et al., 2011), and the complete repertoire transcribed by p53 is increasing.

When cells are not experiencing stress, p53 production occurs at low levels and is continuously inactivated by the E3 ubiquitin ligase MDM-2 (Momand et al., 1992). The importance of the MDM-2/p53 regulatory axis was evidenced after early embryonic lethality in mdm2-null mice and reversion from lethality in double-p53-mdm-2 *knockout* mice (Jones et al., 1995; Montes de Oca Luna et al., 1995). MDM-2 binds to the p53 NTD but rapidly dissociates upon genotoxic stress, ionizing radiation, and UV light leading to p53 activation (Shieh et al., 1997; Saito et al., 2002). After intense stress and irreversible DNA damage, p53 orchestrates a tumor-suppressive program that transcribes genes for pro-apoptotic and senescence proteins to avoid malignant cell transformation.

On the other side, when low levels of stress impact cells that tolerate reparable damage, p53 drives a temporary pro-survival program to aid recovery from the insult. Traditional pro-survival programs include DNA repair, cell-cycle arrest, and antioxidant protein production (Figure 3). In the backstage of these canonical pro-survival programs, p53 works in non-canonical tumor-suppressive programs that include genome stability avoiding aneuploid cells, inhibiting glycolysis and gluconeogenesis, and promoting oxidative phosphorylation to protect cells from metabolic reprogramming (Green and Kroemer, 2009; Vousden and Ryan, 2009; Li et al., 2012; Valente et al., 2013; Kruiswijk et al., 2015) (Figure 3). Alternatively, p53 also represses tumorigenesis by limiting cellular plasticity (Tschaharganeh et al., 2014), inducing autophagy (Maiuri et al., 2010), and non-cell-autonomous activities (Lujambio et al., 2013), damping angiogenesis and metastasis (Figure 3).

## P53-mediated cell death

The first reports linking p53 to apoptosis came from radiation-induced thymocytes (Lowe et al., 1993), colon-derived cell lines (Shaw et al., 1992), and leukemia cells (Yonish-Rouach et al., 1991). Next, p53-dependent apoptosis occurs regardless of transcriptional activity (Caelles et al., 1994). This finding provided the basis for understanding the diversity in which p53 could activate apoptotic signaling. As a transcription factor, p53 was initially found to regulate apoptotic genes. In human vascular smooth muscle cells, p53 can mediate apoptosis through Fas transport to the cell surface from Golgi cytoplasmic stores (Bennett, 1998). In irradiated thymocytes, a fraction of active p53 in the mitochondria directly induces outer membrane permeabilization by forming p53-BclXL complexes and cytochrome *c* release (Mihara et al., 2003; Chipuk et al., 2004). Further, the cytosolic localization of endogenous p53 was necessary and sufficient to trigger apoptosis (Chipuk et al., 2004).

The cooperativity in which all four DNA-binding domains (DBD) stick into the DNA is an additional aspect of tuning the p53 cell death response (Schlereth et al., 2010; Timofeev et al., 2013). Mutations weakening the strength of the inter-dimer association caused a substantial reduction of p53 binding to proapoptotic gene promoters. On the contrary, mutants with increased strength led p53 to bind more robust to these proapoptotic targets (Schlereth et al., 2010; Timofeev et al., 2013).

Besides apoptosis, p53 is involved in other cell death mechanisms such as necrosis and ferroptosis. In ischemia-associated oxidative stress, p53 accumulates in the mitochondrial matrix and interacts with cyclophilin D, leading to the permeability transition pore opening that culminates in necrosis (Vaseva et al., 2012). In the case of ferroptosis, acetylation-defective p53 mutants inhibit the cystine uptake by repressing the cystine/glutamate antiporter SLC7A11, a regulatory axis engaged when cell-cycle arrest, senescence, and apoptosis fails after stress triggered by reactive oxygen species (Jiang et al., 2015).

Autophagy is a self-eating process regulated by p53 that culminates either in pro-survival or pro-death cascades (Mizushima and Komatsu, 2011). Autophagy is constitutive in most cells and operates under physiological circumstances to recycle macromolecules and support cellular metabolism. Under stress, such as hypoxia or nutrient depletion, p53-deficient cancer cells enhance autophagy to maintain ATP levels, improving survival. Moreover, cytoplasmic but not nuclear p53 dampens autophagy in p53-null cells (Tasdemir et al., 2008). Contrastingly, p53 regulates pro-autophagic proteins such as DRAM, a p53 target gene encoding a lysosomal protein related to p53-mediated apoptosis (Crighton et al., 2006). Other p53 target genes such as PUMA and Bax participate in autophagy of the mitochondria that culminates in apoptosis (Yee et al., 2009).

In *Drosophila*, cell competition helps the growth of healthy tissue and regulates tissue size. Confrontation of wild-type cells and cells overexpressing Myc activity is p53-dependent in which p53 senses conflict and endows a genetic program to support a supercompetitor status and transmission of killing signals for weaker cells (de la Cova et al., 2014). The nuances of p53 regulating cell death or survival are deeply explored within tumor development. However, p53 goes beyond cellular decisions and participates in life perpetration mechanisms such as maternal reproduction and blastocyst implantation (Hu et al., 2007). The leukemia inhibitory factor (LIF) is a multi-functional cytokine highly expressed in uterine endometrium before blastocyst implantation (Cullinan et al., 1996). Low levels of LIF are associated with deficient implantation and infertility in humans. The LIF gene has an intronic DNA-binding element in which p53 is bound, regulating LIF transcriptional activity in mice and humans (Hu et al., 2007). Notably, the most common single nucleotide polymorphism (SNP) within the TP53 gene occurs at codon 72, leading to an Arg-to-Pro substitution (R72P). It is worth mentioning that the R72 p53 has higher transcriptional activity toward LIF expression than P72, and Caucasian women carrying P72 p53 showed infertility after *in vitro* fertilization trials, providing a link that p53 may regulate human reproduction (Kay et al., 2006).

## Metabolic roles of p53

Cells under physiological conditions prefer efficient rather than fast energy production, which means 36 molecules of ATP per glucose molecule during oxidative phosphorylation instead of two during glycolysis. In contrast, cancer cells adopt aerobic glycolysis (Warburg effect), a fast but inefficient strategy for energy production. P53 regulates several aspects avoiding the metabolic reprogramming of cancer cells. Nonetheless, cancer-related p53 gain-of-function mutations have been shown to promote the Warburg effect by inducing the translocation of GLUT1 receptors to the plasma membrane (Zhang et al., 2013). Also, cancer-related p53 mutations increase the pentose phosphate flux by avoiding p53 binding to glucose-6-phosphate dehydrogenase (G6PD), the rate-limiting enzyme of the pentose phosphate pathway (PPP) (Jiang P. et al., 2011). The absence of p53-G6PD interaction increases glucose consumption, shifting to macromolecules’ production via the PPP flux. Therefore, abnormal p53 contributes to the Warburg effect and enhanced biosynthesis in cancer cells (Jiang P. et al., 2011). During hypoxia, p53 deficiency induces the expression of monocarboxylate transporter 1 (MCT1), contributing to the efflux of lactate in cancer cells (Boidot et al., 2012). On the contrary, active p53 binds to the promoter of MCT1, altering the MCT1 mRNA stabilization that represses MCT1 protein levels. This mechanism illustrates a metabolic adaptation mediated by p53 to adjust lactate levels depending on cancer cells presenting more oxidative or glycolytic status (Boidot et al., 2012). Another way p53 avoids lactate production is by decreasing the levels of pyruvate dehydrogenase kinase 2 (PDK2), a gatekeeper of the pyruvate conversion to acetyl-CoA. PDK2 phosphorylates the pyruvate dehydrogenase complex (PDH), inhibiting the enzyme activity. By decreasing PDK2, p53 helps maintain PDH activity that diverts pyruvate to acetyl-CoA instead of lactate, a downregulatory condition of the Warburg effect (Contractor and Harris, 2012).

**FIGURE 4:**
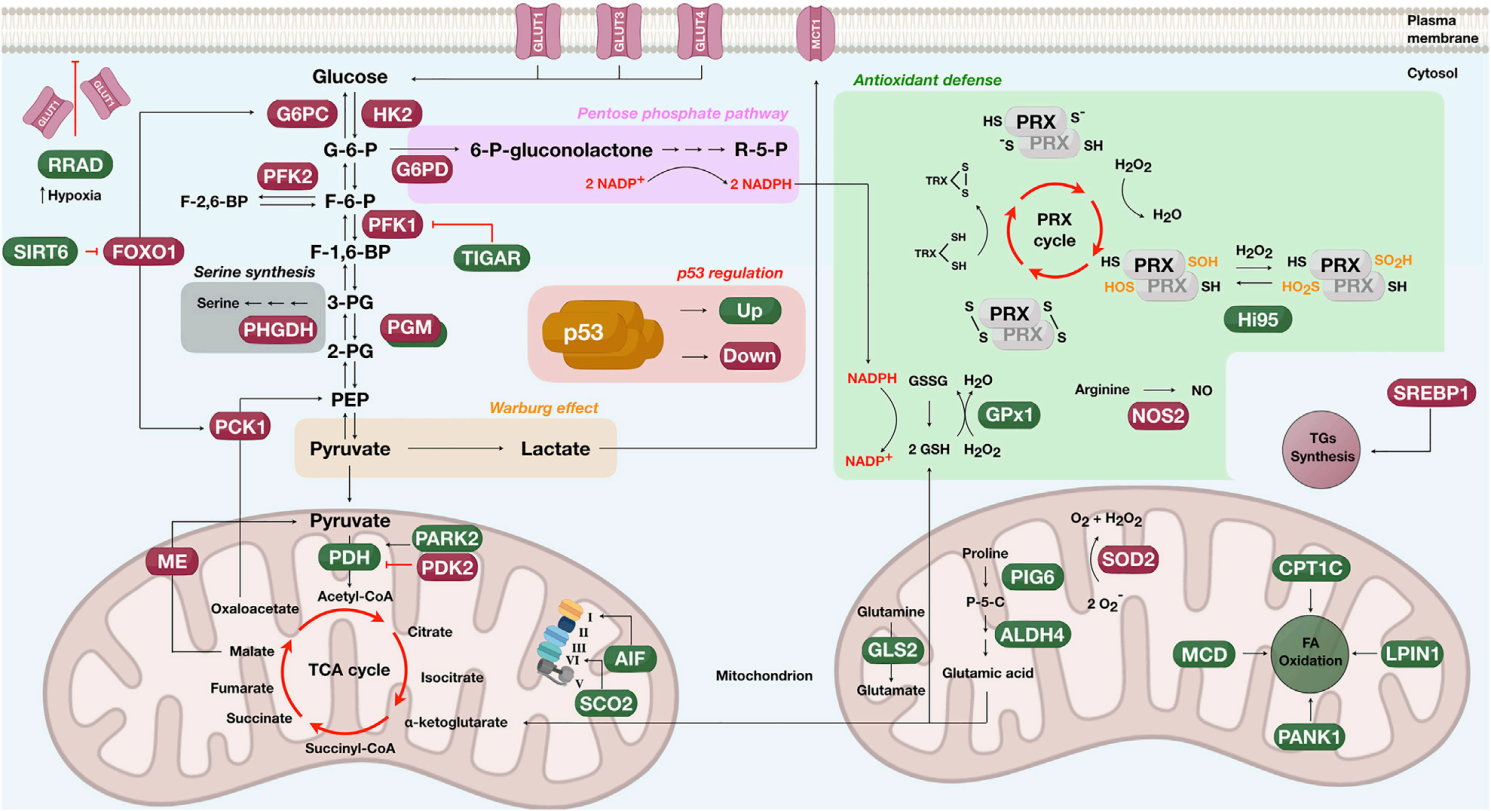
Schematics showing the participative role of p53 in metabolic pathways such as the glycolysis (glucose to pyruvate), the oxidative phosphorylation (left mitochondrion), the pentose phosphate (pink rectangle), the Warburg effect (orange rectangle), the serine synthesis (gray rectangle), proline and glutamine degradation (right mitochondrion), the antioxidant defense (green box), the fatty acid (FA) oxidation (right mitochondrion), and triglycerides (TGs) synthesis (cytosol). Proteins upregulated by p53 are shown in green and downregulated in dark red. G-6-P, glucose-6-phosphate; F-6-P, fructose-6-phosphate; F-1,6-BP, fructose-1,6-biphosphate; F-2,6-BP, fructose-2,6-biphosphate; 3-PG, 3-phosphoglycerate; 2-PG, 2-phosphoglycerate; PEP, phosphoenolpyruvate; R-5-P, ribose-5-phosphate; P-5-C, pyrroline-5-carboxylate; TCA, tricarboxylic acid; PRX, peroxiredoxins; TRX, thioredoxin; GSH, glutathione; GSSG, oxidized glutathione; -SH, sulfhydryl group; S-S, oxidized sulfhydryl (disulfides); NO, nitric oxide; -SOH, sulfenic acid; -SO_2_H, sulfinyl group; O_2_^−^, superoxide radical; H_2_O_2_, hydrogen peroxide; NADP+, nicotinamide adenine dinucleotide phosphate; NADPH, reduced form of nicotinamide adenine dinucleotide phosphate. For protein names controlled by p53, please check in-text citations.

Several studies show wild-type p53 modulating glycolytic enzymes to decrease the glycolytic flux. Other proteins indirectly repress glycolysis through intermediate metabolites or glucose uptake (Schwartzenberg-Bar-Yoseph et al., 2004) (Figure 4). P53 suppresses the transcriptional activity of GLUT1 and GLUT4 receptors in a tissue-specific manner, reducing the glucose intake and subsequently the glycolytic flux (Schwartzenberg-Bar-Yoseph et al., 2004). During hypoxia, p53 upregulates a Ras-related small GTPase called RRAD and represses glycolysis by inhibiting GLUT1 translocation into the plasma membrane (Zhang C. et al., 2014). By limiting the activities of IkΒ kinase α and β (IKKα and IKKβ), p53 modulates the nuclear factor-kB (NF-kB) pathway and dampens the expression of GLUT3 (Kawauchi et al., 2008). The glycolytic enzyme, hexokinase 2 (HK2), converting glucose to glucose-6-phosphate, is also a p53 target gene (Mathupala et al., 1997; Wang L. et al., 2014). For example, insights from PTEN-/p53-deficient mice fibroblasts revealed upregulation of HK2 in prostate cancer cells (Wang L. et al., 2014).

On a sidearm of glycolysis, fructose-6-phosphate (F-6-P) is phosphorylated by phosphofructokinase 2 (PFK2) to generate fructose-2,6-biphosphate (F-2,6-BP), a metabolite that allosterically activates PFK1 to stimulate glucose breakdown. P53 binding to the PFK2 promoter induces transcriptional repression and diminishes PFK2 protein levels (Franklin et al., 2016; Ros et al., 2017). Another example limiting the glycolytic flux is the expression of a p53 target gene called TIGAR. The protein encoded by the TIGAR gene decreases the fructose-2,6-biphosphate and reactive oxygen species (ROS), controlling glycolysis (Bensaad et al., 2006). Parkin (PARK2), a Parkinson’s disease-associated gene, also aids p53 in glucose metabolism. PARK2 deficiency induces glycolysis and reduces mitochondrial respiration (Zhang et al., 2011), and PARK2 expression upregulates pyruvate dehydrogenase (PDH) by increasing E1α1, an essential component of the pyruvate dehydrogenase complex (Zhang et al., 2011). Phosphoglycerate mutase (PGM) is also a p53 target, but modulation seems to occur in a tissue-dependent manner. For instance, results from mouse fibroblasts have shown that p53 down-regulates PGM (Kondoh et al., 2005). In contrast, results from muscle cells revealed the opposite, with p53 activating PGM and promoting glycolysis (Ruiz-Lozano et al., 1999).

While controlling the glycolytic rate, p53 also drives oxidative phosphorylation (Figure 4). P53 balances the oxidative and glycolytic routes by upregulating the cytochrome *c* oxidase 2 (SCO2) transcription, the critical regulator of the cytochrome *c* oxidase (COX) complex IV. Disruption of the SCO2 gene in cancer cells expressing wild-type p53 rescued the metabolic program toward glycolysis (Matoba et al., 2006). The AIF protein is a p53 target gene in the inner mitochondrial membrane presenting NADH oxidase activity. The upregulation of AIF by p53 sustains the mitochondrial complex I integrity and the oxidative phosphorylation system (Stambolsky et al., 2006). Another regulatory axis considers the amount of p53 protein levels. Cells carrying decreased levels of CPEB, a protein promoting 3′ RNA polyadenylation, have fewer mitochondria, reduced respiration, and enhanced glycolysis. These events are accompanied by p53 mRNA having a short poly(A) tail and reduced translational efficiency, which causes p53 protein reduction (Burns and Richter, 2008). The link between p53 regulating metabolic enzymes and senescence was unraveled by showing that p53 represses malic enzymes 1 and 2, catalyzing the oxidative decarboxylation of malate to produce pyruvate. By inhibiting malic enzymes, p53 regulates cell metabolism, proliferation, and the outcome in which the senescence response is achieved (Jiang et al., 2013). Another interesting finding is that α-ketoglutarate, a metabolite of the tricarboxylic acid cycle, works as an effector of p53 tumor-suppressive activities (Morris et al., 2019).

Besides downregulating the glycolytic flux and promoting oxidative phosphorylation, p53 also impacts gluconeogenesis, a metabolic pathway generating glucose from non-carbohydrate precursors (Figure 4). P53 represses two rate-limiting enzymes of gluconeogenesis, the phosphoenolpyruvate carboxy-kinase (PCK1) and glucose-6-phosphatase (G6PC) (Zhang P. et al., 2014). The mechanism under which p53 tunes down PCK1 and G6PC is by activating SIRT6, a histone deacetylase acting on the nuclear extrusion of the forkhead box protein O1 (FoxO1), the critical transcription factor mediating PCK1 and G6PC expression.

Another p53 tumor-suppressive function linked to the metabolic response is the antioxidant program to avoid genome oxidation by ROS (Sablina et al., 2005) (Figure 4). Antioxidant genes regulated by p53 include sestrins, NOS2, ALDH4, GLS2, GPx1, and MnSOD (Forrester et al., 1996; Budanov et al., 2004; Hussain et al., 2004; Yoon et al., 2004; Suzuki et al., 2010). In the case of Sestrins, these enzymes act as cysteine sulfinyl reductases, reverting inactive overoxidized peroxiredoxins (Prxs), the enzymes that metabolize peroxides. By reducing Prxs, sestrins re-establish the cell’s antioxidant surveillance of peroxides, avoiding oxidative stress (Budanov et al., 2004).

The p53 is also participative in proline degradation by regulating ALDH4 expression, a glutamate-γ-semialdehyde dehydrogenase that protects intracellular ROS generation (Yoon et al., 2004) (Figure 4). ALDH4 irreversibly converts the intermediate of proline degradation, pyrroline-5-carboxylate (P-5-C), into L-glutamic acid, exhausting the proline pool. The first enzyme of the proline cycle is proline oxidase which catalyzes the conversion of proline to P-5-C with the donation of electrons to cytochrome *c*. This reaction supports ROS formation by creating reducing potential. P-5-C is either recycled to proline by P5C reductase or transformed into L-glutamic acid by ALDH4, ending the reducing cycle (Yoon et al., 2004). It is worth mentioning that p53 upregulates either antioxidant proteins, such as ALDH4, or pro-oxidant proteins, such as the proline oxidase PIG 6 (Donald et al., 2001; Rivera and Maxwell, 2005). Whereas pro-oxidant proteins may help p53 induce cell death, antioxidant proteins would assist cellular adaptation against insults. A list of other p53 target genes adopting controversial roles is presented in other reviews (Kruiswijk et al., 2015).

An additional p53 regulatory axis occurs through glutaminase 2 (GLS2), supporting the flux of mitochondrial respiration and the antioxidant defense program (Figure 4). GLS2 hydrolyzes glutamine to glutamate and is a p53 target gene during stress and non-stress circumstances (Hu et al., 2010b; Suzuki et al., 2010). GLS2 favors mitochondrial respiration and ATP generation by increasing the production of glutamate and α-ketoglutarate. It also increases reduced glutathione (GSH) levels, leading to ROS deactivation and attenuating oxidative stress (Hu et al., 2010b; Suzuki et al., 2010). A solution to deactivate ROS is glutathione peroxidase (GPx1), another enzyme upregulated by p53 (Tan et al., 1999; Hussain et al., 2004). GPx1 uses GSH to reduce H_2_O_2_ to water at the cost of producing GSSG.

Another reactive molecule is the superoxide anion. The generation of superoxide anion (O_2_^−^) inside the mitochondrion occurs when electrons leak from oxidative phosphorylation and reduce diatomic oxygen (O_2_). Manganese superoxide dismutase (SOD2) converts two molecules of superoxide anion into atomic oxygen and H_2_O_2_. SOD2 is commonly deregulated in human cancer, and several reports provide evidence of p53 downregulating SOD2 to induce cell death (Pani et al., 2000; Drane et al., 2001; Hussain et al., 2004). Besides the role of p53 in avoiding ROS generation, it also suppresses reactive nitrogen species (RNS) such as nitric oxide (NO). P53 binds to the promoter of NO synthase 2 (NOS2), downregulating the protein levels of NOS2 and ultimately the excessive production of NO (Forrester et al., 1996; Ambs et al., 1998).

P53 also participates in lipid metabolism, regulating critical transcription factors and enzymes to promote fatty acid oxidation and suppress lipid biosynthesis (Figure 4), commonly observed in cancer cells (Kruiswijk et al., 2015; Liu et al., 2019a). For example, the transcriptional program of p53 in obese mice leads to the downregulation of the sterol regulatory element-binding protein 1 (SREBP1), a transcriptional regulator of triglyceride (TG) synthesis and lipogenic enzymes (Yahagi et al., 2003). While repressing lipid anabolism, p53 promotes fatty acid (FA) oxidation by upregulating Lpin1 (Assaily et al., 2011), CPT1C (Sanchez-Macedo et al., 2013), MCD (Liu et al., 2014), and PANK1 (Wang et al., 2013). Lpin1 is a bifunctional protein acting as a nuclear transcriptional coactivator to regulate genes involved in FA oxidation. Upon glucose deprivation, Lpin1 is expressed in a p53-dependent manner in mouse myoblasts through ATM phosphorylation at p53 Ser-18 (Assaily et al., 2011). Another upregulated p53 target gene is the pantothenate kinase-1 (PANK1), an enzyme that catalyzes the rate-limiting reaction of CoA production. PANK1 potentially facilitates FA oxidation as PANK1-*knockout* mice present β-oxidation and gluconeogenesis disruption (Wang et al., 2013). In the case of MCD, for proper expression of malonyl-CoA decarboxylase (MCD) to control FA oxidation, a functional regulatory axis comprising p53-MDM2 and ribosomal proteins is required (Liu et al., 2014).

Finally, p53 has shown regulatory involvement in a glycolysis-diverting pathway that generates serine, a necessary amino acid for anabolic pathways such as nucleotide, glutathione, phospholipids, and other amino acids (Amelio et al., 2014) (Figure 4). The role of serine in oncogenesis is that serine works as a hub for biosynthetic pathways required for tumor growth, and it also disrupts cells’ antioxidative abilities (Amelio et al., 2014). In breast cancer, enzymes linked to serine production are highly expressed (Possemato et al., 2011). In melanoma cells, after serine starvation, p53 downregulates the first enzyme of serine production, phosphoglycerate dehydrogenase (PHGDH), leading to an apoptotic response (Ou et al., 2015). Indeed, cancer cells require p53 to handle serine starvation, adjusting the metabolic program to an antioxidative response (Maddocks et al., 2013).

In conclusion, p53 tunes metabolism by multiple strategies: 1) direct protein binding to control glucose uptake (e.g., GLUT receptors) or the rate-limiting enzyme of the pentose phosphate pathway (e.g., G6PDH), 2) augmenting transcriptional activity modulating gluconeogenesis (e.g., SIRT6), glycolysis (e.g., TIGAR), and oxidative phosphorylation (e.g., PDH, SCO2, and AIF) or 3) repressing transcriptional activity and allosteric activators such as F-2,6-BP or glycolytic enzymes (e.g., HK2, PFK1, PFK2, and PGM). Further, the mammalian target of rapamycin (mTOR), a conserved serine/threonine kinase integrating multiple cellular signals for anabolic and catabolic processes, has been linked to the p53 regulatory axis (Cui et al., 2021). Active p53 delivers a personalized transcriptional program and binds to specific partners upon cellular insults. For p53 to adjust physiological metabolism and deal with metabolic reprogramming in cancer, discovering p53 functional oligomeric states (p53-HOS) inside the nuclei, cytosol, and mitochondrion might represent a plausible explanation of how p53 copes with such a multiverse of activities.

## The p53 gain-of-function activity

Cell lines lacking endogenous p53, when transformed with p53 tumor-associated mutations, revealed growth advantage and higher expression of multidrug resistance genes compared with the parental line (Dittmer et al., 1993). When compared to heterozygous (p53^+/−^) or null (p53^−/−^), mutant p53 (p53^+/Mut^) mice developed osteosarcomas and carcinomas that metastasized at a higher frequency, increased tumor proliferation, and presented a cooperative action with activated *ras* in cell transformation (Lang et al., 2004; Olive et al., 2004)*.* This event suggests altered cellular signaling mediated by mutant p53. Innumerous efforts have indicated a broad spectrum of mutant p53 GoF outcomes, including cell invasion, migration, angiogenesis, survival, proliferation, and tissue remodeling (Muller and Vousden, 2013).

Hotspot mutations (including R175, G245, R248, R249, R273, and R282) lie within the DBD—denoting that DNA interaction and its intrinsic metastability constitutes one aspect of understanding GoF and LoF effects. Although clustered in the same domain, hotspot mutations impair DNA interaction or disrupt critical contacts responsible for correct protein folding (Muller and Vousden, 2013). Accumulating evidence has suggested that protein-protein interaction between mutant p53 and other cellular proteins could contribute to the GoF. For instance, mutant p53 interactions with p63 and p73 are disrupted, and the p73 DNA binding activity is impaired when p53 mutants are co-translated *in vitro* (Marin et al., 2000; Gaiddon et al., 2001).

Further, the p53 mutations R248W and R273H disrupt the response of double-stranded DNA breaks by affecting the recruitment of the Mre11–Rad50–NBS1 (MRN) complex to the site of DNA damage, leading to genetic instability (Song et al., 2007). Further, exploring the p53 network, a correlation between mutant p53 and prolyl isomerase (Pin1) was proposed (Girardini et al., 2011). In cancer, p53 is phosphorylated on sites recognized by Pin1, and the Pin1/mutant p53 axis induces a set of genes related to malignant phenotypes (Girardini et al., 2011). In lung carcinoma, Muller and colleagues explored what causes cells expressing mutant p53 to migrate randomly and increase invasiveness. Integrin α5β1 and p53 are interconnected pathways promoting cell growth in normal conditions but are disrupted in the presence of mutant p53 (Muller et al., 2009). Surprisingly, the inhibition of the α5β1 integrin and EGFR reverted the migratory behavior, pointing out a possible target for further investigation (Muller et al., 2009).

The steroids biosynthesis pathway was downregulated in 3D cell cultures expressing mutant p53 (Freed-Pastor et al., 2012). Although it is an essential pathway in cholesterol biosynthesis—which maintains membrane integrity and cell division—some intermediates execute vital functions such as PTMs of Ras and RhoA. Interestingly, genes related to the mevalonate axis are overexpressed in both 3D cell cultures expressing mutant p53 and human breast cancer patients (Freed-Pastor et al., 2012). Different p53 mutations indeed display variable outcomes depending on the cellular content. p53 gains function by increasing and prolonging the response of epithelial cells to low amounts of inflammatory cytokine, reinforcing a chronic state of NF-kB activation. p53 accumulates in cellular regions, fueling the inflammatory process and tissue damage, which leads to invasive carcinoma (Cooks et al., 2013). In this context, the structural nature of accumulated p53 would be valuable in bringing mechanistic insight into the disease phenotype.

It is essential to emphasize that the p53 transactivation domain remains intact in hotspot mutations. However, the binding of wild-type p53 to gene-proximal regions (less than 10 kb) of transcription start sites (TSS) strongly differs from the mutant p53 binding (Zhu et al., 2015). This event could be explained by association with distinct protein partners as p53 mutants target specific chromatin regulators not observed in the wild-type protein (Zhu et al., 2015).

Further, the knockdown of chromatin regulators in cancer cells expressing mutant p53 led to a dramatic loss of cell growth (Zhu et al., 2015). Wiech et al. have shown that HSP70, a molecular chaperone, stabilizes mutant p53 and inhibits p53 degradation (Wiech et al., 2012). Recently, mutant p53 but not the wild-type p53 constituted extracellular vesicles (EVs) (Ma et al., 2021). Rising evidence demonstrates that EVs could promote a pro-tumor microenvironment, transporting mRNAs, DNA, and even proteins (Jeppesen et al., 2019). The authors stated that mutant p53 spread to the stromal compartment through EVs leading to tumor growth. Interestingly, this phenotype is inhibited by HSP90 small inhibitors, suggesting a pivotal role in p53 traffic (Ma et al., 2021). A reasonable mechanism in which p53 accomplishes a diverse set of GoF activities would involve mutant p53 forming functional HOS. Indeed, mutant p53 oligomers and aggregates have been shown to participate in drug resistance, a typical GoF phenotype in cancer (Yang-Hartwich et al., 2015; Pedrote et al., 2020).

## Conclusion

Back in the drama “Man of a Thousand Faces,” a silent movie exploring the life of Lon Chaney and his abilities to transform himself using makeup techniques, the mutant p53 GoF phenotype is wide enough to interpret p53 as a protein of a thousand faces analogously. Part of the makeup techniques p53 uses to enter the stage include a plethora of PTMs at the NTD and CTD. Once affected by some mutations, p53 undergoes a higher tendency to form HOS, another makeup style allowing p53 malignant transformation in cancer. The diversity of p53 characters earned p53 multiple interpretations as in an apoptotic show or during a metabolic adaptation rehearsal. The use of cryo-EM, nuclear magnetic resonance, and X-ray diffraction are shining light to visualize and unravel the whole p53 makeup repertoire. The correlation between GoF and pro-tumorigenic action is clear. However, it is essential to emphasize that p53 is a key actor of several cellular pathways and that distinct mutations would generate particular outcomes that deserve to be visited. Let’s continue unraveling the p53 show!

## Glossary

Cryo-EM: cryo-electron microscopy
CTD: C-terminal domain
DBD: DNA-binding domain
ERKs: extracellular signal-regulated protein kinases
F-2,6-BP: fructose-2,6-biphosphate
F-6-P: fructose-6-phosphate
FA: fatty acid
G6PC: glucose-6-phosphatase
G6PD: glucose-6-phosphate dehydrogenase
GLS2: glutaminase 2
GoF: gain-of-function
GPx1: glutathione peroxidase
GSH: glutathione
GSSG: oxidated glutathione
HHP: high hydrostatic pressure
HK2: hexokinase 2
HOS: higher-order structures
LLPS: liquid-liquid phase separation
LoF: loss-of-function
MCD: malonyl-CoA decarboxylase
MCT1: monocarboxylate transporter 1
NF-kB: nuclear factor-kB
NMR: nuclear magnetic resonance
NO: nitric oxide
NOS2: NO synthase 2
NTD or TAD: N-terminal transactivation domain
OD or TET: oligomerization domain
p53-DBD: p53 DNA-binding domain
p53-HOS: p53 higher-order structures
P-5-C: pyrroline-5-carboxylate
PANK1: pantothenate kinase-1
PARK2: Parkin
PCK1: phosphoenolpyruvate carboxy-kinase
PDH: pyruvate dehydrogenase complex
PDK2: pyruvate dehydrogenase kinase 2
PGM: phosphoglycerate mutase
PHGDH: phosphoglycerate dehydrogenase
Prxs: peroxiredoxins
PTMs: post-translational modifications
RE: responsive elements
RNS: reactive nitrogen species
ROS: reactive oxygen species
SCO2: cytochrome *c* oxidase 2
SOD2: superoxide dismutase
SREBP1: regulatory element-binding protein 1
TG: triglyceride
UPR: unfolded protein response
